# Chronic hypoxia disrupts T regulatory cell phenotype contributing to the emergence of exTreg-T_H_17 cells

**DOI:** 10.3389/fphys.2023.1304732

**Published:** 2024-01-29

**Authors:** Benjamin J. Lantz, Mika Moriwaki, Olufunmilola M. Oyebamiji, Yan Guo, Laura Gonzalez Bosc

**Affiliations:** ^1^ Gonzalez Bosc Laboratory, Health Sciences Center, Cell Biology and Physiology, University of New Mexico, Albuquerque, NM, United States; ^2^ Division of Molecular Medicine, Health Sciences Center, Internal Medicine, University of New Mexico, Albuquerque, NM, United States; ^3^ Department of Public Health and Sciences, University of Miami, Miami, FL, United States

**Keywords:** chronic hypoxia, Treg, T_H_17, exTreg, CH-induced PH, immune imbalance, exTreg-T_H_17, pulmonary inflammation

## Abstract

The imbalance between pro-inflammatory T helper 17 (T_H_17) cells and anti-inflammatory regulatory T cells (Tregs) has been implicated in multiple inflammatory and autoimmune conditions, but the effects of chronic hypoxia (CH) on this balance have yet to be explored. CH-exposed mice have an increased prevalence of T_H_17 cells in the lungs with no change in Tregs. This imbalance is significant because it precedes the development of pulmonary hypertension (PH), and T_H_17 cells are a major contributor to CH-induced PH. While Tregs have been shown to attenuate or prevent the development of certain types of PH through activation and adoptive transfer experiments, why Tregs remain unable to prevent disease progression naturally, specifically in CH-induced PH, remains unclear. Our study aimed to test the hypothesis that increased T_H_17 cells observed following CH are caused by decreased circulating levels of Tregs and switching of Tregs to exTreg-T_H_17 cells, following CH. We compared gene expression profiles of Tregs from normoxia or 5-day CH splenocytes harvested from Foxp3^tm9(EGFP/cre/ERT2)Ayr^/J x Ai14-tdTomato mice, which allowed for Treg lineage tracing through the presence or absence of EGFP and/or tdTomato expression. We found Tregs in CH exposed mice contained gene profiles consistent with decreased suppressive ability. We determined cell prevalence and expression of CD25 and OX40, proteins critical for Treg function, in splenocytes from Foxp3^tm9(EGFP/cre/ERT2)Ayr^/J x Ai14-tdTomato mice under the same conditions. We found T_H_17 cells to be increased and Tregs to be decreased, following CH, with protein expression of CD25 and OX40 in Tregs matching the gene expression data. Finally, using the lineage tracing ability of this mouse model, we were able to demonstrate the emergence of exTreg-T_H_17 cells, following CH. These findings suggest that CH causes a decrease in Treg suppressive capacity, and exTregs respond to CH by transitioning to T_H_17 cells, both of which tilt the Treg–T_H_17 cell balance toward T_H_17 cells, creating a pro-inflammatory environment.

## Introduction

Chronic hypoxia (CH) drives a number of physiological changes, including polycythemia and thickening of pulmonary arteries through increased proliferation of the surrounding smooth muscle cells, as well as cell hypertrophy, enhanced pulmonary vasoreactivity, and inflammation, all of which contribute to increased pulmonary pressure (Shimoda et al., 2000; Bierer et al., 2011; Shimoda and Laurie, 2013; Jernigan and Resta, 2014; Maston et al., 2017). T helper 17 (T_H_17) cells are a major group of pro-inflammatory immune cells that drive inflammation (Martinez et al., 2008; Yang et al., 2008; Yan et al., 2020). Maston et al. (2017) demonstrated the role of T_H_17 cells in CH-induced pulmonary hypertension (PH), finding localization of T_H_17 cells around pulmonary arteries in CH-exposed mice and an increased prevalence of T_H_17 cells in the lungs with no change in regulatory T cells (Tregs) (Maston et al., 2017). Furthermore, in the same report, our group showed that inhibiting T_H_17 cells attenuated indices of CH-induced PH and increased the proportion of Tregs while decreasing T_H_17 cells in the lungs. Finally, adoptive transfers of polyclonal T_H_17 cells resulted in spontaneous development of PH, recapitulating CH-induced PH (Maston et al., 2017). These prior findings suggest that an imbalance between T_H_17 cells and Tregs plays a role in the pathogenesis of CH-induced PH.

Tregs act in an anti-inflammatory manner, often opposing the pro-inflammatory effects of T_H_17 cells, preventing excessive immune responses, which averts a prolonged inflammatory state that can lead to autoimmunity (Bluestone and Abbas, 2003; Schmitt and Williams, 2013; Lee, 2018; Yan et al., 2020). The imbalance between Tregs and T_H_17 cells has been implicated in the pathogenesis of various cancers and autoimmune diseases, with the severity of the imbalance correlating with disease outcomes (Komatsu et al., 2014). In pancreatic and non-small-cell lung cancers, accumulation of Tregs is observed within tumors, preventing effective anti-tumor responses by T_H_17 cells (Gnerlich et al., 2010; Shimizu et al., 2010). In inflammatory bowel disease and rheumatoid arthritis, a decrease in Tregs tilts the balance toward T_H_17 cells, resulting in uncontrolled inflammation and autoimmunity (Rubtsov et al., 2010; Nie et al., 2015; Lee, 2018; Veselinova Velikova et al., 2020; Go et al., 2021). When looking at the suppressive capacity of Tregs, or how well Tregs can suppress an immune response, CD25 and OX40 expression levels have been identified as indicators of Treg distress (So and Croft, 2007; Pedersen and Lauritsen, 2009; Cabrera et al., 2012; Russell et al., 2012; Fu et al., 2020). Tregs with decreased expression of CD25 were found to have a decreased suppressive capacity due to a loss in their ability to respond to IL-2, the signaling of which results in the expression of forkhead box P3 (FoxP3) and CD25 (Sakaguchi et al., 2013; Harris et al., 2023). FoxP3 is the major transcription factor found in Tregs. This phenotype has been identified in Tregs collected from the peripheral blood of rheumatoid arthritis patients in a disease severity-dependent manner (Komatsu et al., 2014; Go et al., 2021; Shevyrev et al., 2021). When looking at OX40, Tregs with elevated levels of OX40 were found to have a less suppressive capacity through the downregulation of IL-10, one of the major anti-inflammatory cytokines released by Tregs (So and Croft, 2007; Fu et al., 2020). The discovery of exTregs, cells that have lost their Treg phenotype, becoming T_H_17 cells, commonly denoted as exTreg-T_H_17 cells, and their role in disease pathogenesis has led to new ideas about how the Treg–T_H_17 cell balance alters and contributes to disease (Zhou et al., 2009; Komatsu et al., 2014). With these studies in mind and building off our previous findings, we hypothesized that the increase in T_H_17 cells, following CH, is due to a reduction in circulating levels of active Tregs and switching of Tregs to exTreg-T_H_17 cells.

## Materials and methods

### Animals

C57B6/J female and male mice that were considered wild types (WTs) were purchased from The Jackson Laboratory, and cells derived from these mice were used as unstained controls in flow cytometry studies. Foxp3^tm9(EGFP/cre/ERT2)Ayr^/J ^ERT2^ (stock #:016961) and Ai14-tdTomato mice (stock #:007914) were obtained from The Jackson Laboratory and crossed in-house. Foxp3^tm9(EGFP/cre/ERT2)Ayr^/J x Ai14-tdTomato mice received five, once per day, i.p. injections of 2 mg of tamoxifen (Sigma-Aldrich) suspended in corn oil (C8267, Sigma-Aldrich), starting between 8 and 12 weeks of age. The recovery period, following tamoxifen injections, was 14 days. Foxp3^tm9(EGFP/cre/ERT2)Ayr^/J x Ai14-tdTomato mice contain an internal ribosome entry site (IRES) and an enhanced green fluorescent protein (EGFP) fused to CreERT2 fusion gene downstream of the internal stop codon of the forkhead box P3 (FoxP3) gene, allowing labeling of Tregs by tdTomato in a tamoxifen-inducible manner. These mice are used to study the lineage stability and genetic fate mapping of Tregs. Il17a^cre^/J (stock #:016879) mice were obtained from The Jackson Laboratory and crossed with Ai14-tdTomato mice in-house, creating the Il17a^cre^/J x Ai14-tdTomato mouse model. This model has Cre recombinase inserted into the Il17a locus (Il17aCre). After being crossed with B6 Ai14-tdTomato fl/J mice, IL-17A-expressing cells expressed the tdTomato reporter. These mice allow the study of lineage stability, genetic fate mapping, and the number of T_H_17 cells. The levels of Cre expression and, therefore, tdTomato mark the active T_H_17 cells (Madisen et al., 2010). Heterozygote mice were used because homozygotes lack IL-17A expression. All mice were kept on standard 12-h day/night cycles, housed in filtered air cages, and fed a regular chow diet.

### Chronic hypoxia exposure

Mice that were exposed to CH were placed in a hypobaric chamber set to 380 mmHg (−18,000 ft elevation) for 5 days. The mice maintained in normoxic conditions were housed in the standard animal room with a pressure of −630 mmHg, which is the atmospheric pressure in Albuquerque, NM. Temperature and humidity were observed and maintained at 23.3°C ± 2.5°C and 30%, respectively, within both settings.

### Splenocyte isolation and preparation

Mice were placed under isoflurane/O_2_ anesthesia, tested for pain responses, euthanized via decapitation, and the spleens were harvested and placed in 1 x PBS on ice before a single cell suspension was obtained. This was achieved by first mincing the spleen and then passing the pieces through a 70-µm mesh filter (229483, CELLTREAT). The suspension of cells was spun at 500 x g for 5 min at 4°C. The pellet was resuspended in 1 mL of red blood cell (RBC) lysis buffer (00-4300-54, eBioscience) for 1–2 min before being quenched with 20 mL of cold 1 x PBS. The suspension was once again spun and washed with 10 mL of 1 x PBS to remove the cells of any residual RBC lysis buffer. Following another spin, the cells were resuspended in 5 mL of cold FACS buffer (2% FBS, 1 x PBS) and placed on ice for cell counting using a hemocytometer.

### Flow cytometry

Following splenocyte counting and viability assessment with trypan blue, 2 × 10^6^ cells were placed in FACS tubes with a total volume of 500 µL and spun for 5 min at 500 × g at 4°C. Cells were then resuspended in 100 µL of FACS buffer and blocked with 1 µL of anti-mouse CD16/CD32 (101330, BioLegend), as well as stained with 1 µL of diluted LIVE/DEAD yellow (L34968, Thermo Fisher Scientific) for 30 min on ice. The tubes were again filled to 500 µL and spun, followed by two washes of 1 mL FACS buffer. Following the last wash, cells were resuspended in 100 µL of FACS buffer and treated with their extracellular antibodies for 1 h on ice, protected from light. All antibodies were anti-mouse: CD3 (Alexa Fluor 750, clone 17A2R, FAB4841RS), CD4 (PerCP, clone GK1.5, 100432), CD25 (BV650, clone PC61, 102038), and OX40 (PE/Cyanine7, clone OX-86, 119416). The tubes were once again filled to 500 µL and washed twice. Cells were then resuspended in 350 µL of FACS buffer and set aside, on ice and protected from light, for analysis. For intracellular staining, cells that were fixed were placed in 1 mL of fixation/permeabilization buffer (00-5523-00, eBioscience) for 45 min, protected from light, on ice. Cells were spun and washed in 1 × permeabilization buffer (00-5523-00, eBioscience) and resuspended in 100 µL of 1 × permeabilization buffer. Cells were then treated with anti-mouse antibodies against FoxP3 (PE/Cyanine5.5, clone FJK-16s, 35-5773-82) and RORγt (BV421, clone Q31-378, 562894) for 30 min, on ice, protected from light. The tubes were filled to 500 µL with 1 × permeabilization buffer, spun, and washed twice with 1 mL FACS buffer. Cells were then resuspended in a final volume of 350 µL and placed on ice, protected from light, and set aside for analysis. Single color controls were created using the above antibodies, except in the case of tdTomato and EGFP. tdTomato required the transfection of a tdTomato-C1 plasmid (#54653, Addgene) into HEK293 cells, and EGFP utilized GFP beads (A10514, Thermo Fisher Scientific). All samples were run on a Cytek Aurora instrument, followed by compensation with FlowJo v10.8.1. Statistical analysis was performed in GraphPad Prism 9 and expressed as mean ± SD. The normality of residuals was tested using the Shapiro–Wilk test at alpha 0.05. All data passed the normality test, supporting the assumption of a normal distribution. Statistical significance was tested at 95% (*p* < 0.05) confidence level using parametric multiple unpaired t-tests with Holm–Šidák correction when data were separated by sex and a parametric unpaired *t*-test without corrections when data were combined.

### Single-cell RNA sequencing sample preparation

Four 6-week-old male Foxp3^tm9(EGFP/cre/ERT2)Ayr^/J ^ERT2^ crossed with Ai14-tdTomato reporter mice received tamoxifen, followed by a recovery, as previously described. Two mice were placed in CH conditions and two were housed under normoxia, as previously described. Following euthanizing and splenocyte isolation, as previously described, cells were resuspended in 10 mL 1 × PBS for counting and viability assessment. The Invitrogen Countess II automated cell counter was used to determine cell counting and viability. The chamber slide was loaded with 10 µL of 1:1 cell suspension in 0.4% trypan blue stain, according to Countess II specifications. After the cell count was determined, a 5-mL aliquot of each sample was made at a concentration of 2 × 10^5^ viable cells/mL in DPBS, according to 10 × Genomics Single Cell Seq specifications for an optimal input concentration of 200 viable cells/µL.

### Single-cell RNA sequencing analysis

The single-cell sequencing data were processed with the conventional pipeline. Seurat (v4.0.5) (Hao et al., 2021) and R (v4.1.0) were both used for downstream analysis. Cells expressing fewer than 200 genes or more than 7,500 genes and with mitochondrial gene expression content greater than 5%, were removed. tdTomato^+^ cells were found using the WPRE sequence that accompanied tdTomato within the animal model. Data were transformed using SCTransform from the Seurat package. Hypoxia samples and normoxic samples were analyzed separately and integrated using Seurat’s standard integration workflow. Principle component (PC) analysis and an elbow plot were used to visualize the variance and select PCs before unsupervised clustering. Clusters were then determined using the FindNeighbors and FindClusters function with default parameters and a sufficient resolution parameter to capture the biological variability. Cell-type prediction was performed using singleR (v1.6.1) against the mouse Immunologic Genome Project (Hafemeister and Satija, 2019). Differences in proportions of immune cell populations between CH and normoxia samples were analyzed using the chi-squared test. The CD4^+^ memory T-cell type was further subclustered. Differential gene expression was determined using the R package DESEq2 (Love et al., 2014). Significant genes were defined by an adjusted *p*-value ≤0.05. ClusterProfiler (v4.0.5) (Wu et al., 2021) was used for functional enrichment analysis. Pathway analysis was performed with DAVID (Sherman et al., 2022) using all DEGs with an adj. *p*-value ≤0.05. GEO Accession Number: GSE219259.

## Results

### Chronic hypoxia alters immune cell populations

**FIGURE 1:**
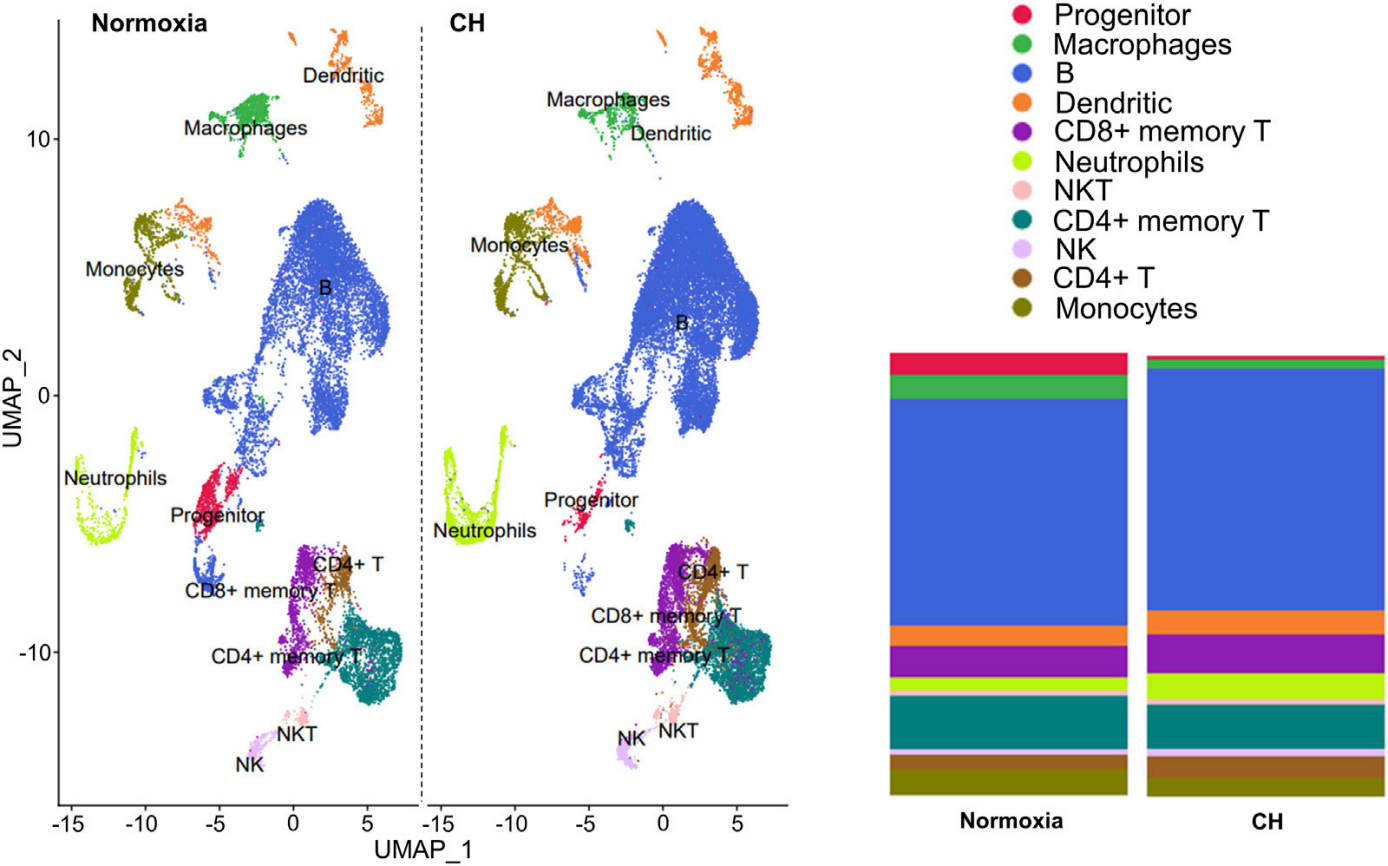
Chronic hypoxia alters immune cell populations. Foxp3^tm9(EGFP/cre/ERT2)Ayr^/J x Ai14-tdTomato male and female mice were exposed to normoxia (*n* = 2) or 5-day CH (*n* = 2). Uniform manifold approximation (UMAP, left) and the proportion of cells per cluster between samples from normoxia and CH-exposed mice (right). Significant differences (*p* < 0.0001) in all immune cell populations except NKT and NK cells between CH and normoxia were determined using the chi-squared test.

The balance between different immune cells is highly regulated in order to maintain a healthy and functional immune system. We investigated the changes that occur in immune cells in splenocytes harvested from Foxp3^tm9(EGFP/cre/ERT2)Ayr^/J x Ai14-tdTomato mice exposed to normoxia or 5-day CH using scRNA-seq. Unbiased cluster analysis of cells from normoxia and CH-exposed mice revealed 11 major immune cell clusters annotated using the Immunologic Genome Project. The results are shown as uniform manifold approximation (UMAP, left), and the proportion of cells per cluster (right) between samples from normoxia and CH-exposed mice was significantly different (Figure 1). To understand the functional implications of the differentially expressed genes (DEGs) between CH-exposed and normoxic mice in the different immune populations (Supplementary Table), we performed Gene Ontology (GO) pathway enrichment analysis in the identified clusters. The analysis revealed multiple shared enriched GO pathways among CD4^+^ memory T cells, CD4^+^ T cells, CD8^+^ memory T cells, and B cells, with the most significant being cytoplasmic translation, oxidative phosphorylation, protein synthesis, and purine nucleotide biosynthesis (Supplementary Table). Innate (macrophages, monocytes, dendritic cells, NK, and NKT cells) and progenitor cells also show enrichment in the same GO pathways (Supplementary Table). The neutrophil cluster was unique since the top enriched pathways were related to B-cell activation and receptor signaling, cell morphogenesis, endocytosis, and antigen binding, while the pathways found to be enriched in T, B, and other innate cells were not enriched in neutrophils (Supplementary Table).

### Chronic hypoxia alters the Treg gene expression profile

**FIGURE 2:**
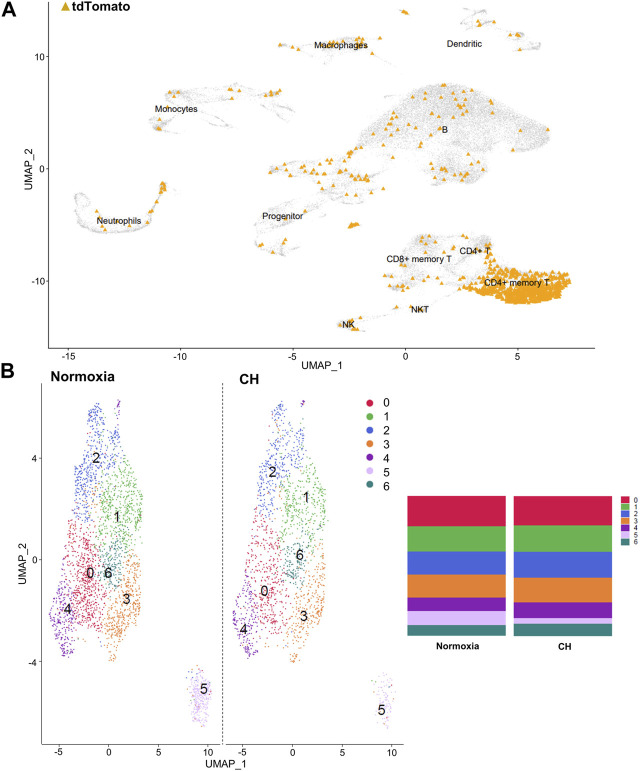
Tregs predominate in the CD4^+^ memory T-cell cluster, and the cluster can be separated into seven distinct clusters. **(A)** Combined UMAP of splenocytes from Foxp3^tm9(EGFP/cre/ERT2)Ayr^/J x Ai14-tdTomato mice exposed to normoxia (*n* = 2) and 5-day CH (*n* = 2). tdTomato sequence depicted in orange within each cluster of cells. **(B)** UMAP display of differences between hypoxia and normoxia subclusters within the CD4^+^ memory T-cell cluster. Bar graphs show the total cell population divided by the percentage of each cell type.

**FIGURE 3:**
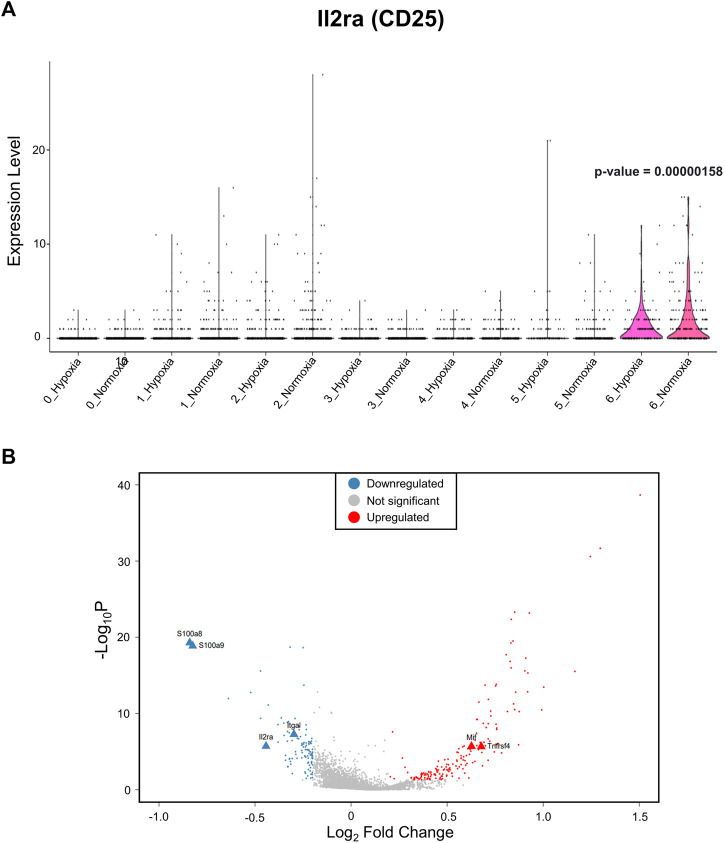
The Treg subcluster shows DEGs associated with the Treg suppressive capacity, following CH. **(A)** Treg marker *Il2ra*, which codifies for CD25, is enriched in subcluster 6. Therefore, this cluster was designated as Tregs. A *p*-value of 0.00000158 when comparing subcluster 6 *Il2ra* expression between normoxia and CH was found. **(B)** Differentially expressed gene (DEG) profile of the T-cell subcluster consistent with Tregs with a reduced suppressive capacity. Blue = downregulated genes, red = upregulated genes, and gray = no significant change (adj. *p*-value<0.05).

After identifying the major immune cell populations, we took advantage of the Foxp3^tm9(EGFP/cre/ERT2)Ayr^/J x Ai14-tdTomato model, which allows lineage tracing of Tregs by marking cells that currently express FoxP3 (EGFP^+^) or, at one point in their life, had FoxP3 expression (tdTomato^+^). To best determine the cluster containing Tregs and exclude other cells known to produce some level of FoxP3, we determined the clusters in which the tdTomato sequence was enriched, finding the vast majority to be contained in the CD4^+^ memory T-cell cluster (Figure 2A). The cells within the CD4^+^ memory T-cell cluster could be further divided into seven subclusters by unsupervised clustering (Figure 2B). Of the seven subclusters of CD4^+^ memory T cells, subcluster 6 had enriched levels of *Il2ra*, the gene coding for CD25, which is predominantly found in Tregs (Figure 3A). We could not use FoxP3 or EGFP to identify Tregs because the transcript counts did not meet the inclusion threshold. Cells found in subcluster 6 were affected by CH exposure. When looking at DEGs within the Treg subcluster 6, we found many significantly altered genes, including multiple genes known to affect the Treg suppressive capacity, including *Il2ra* (downregulated) and *Tnfrsf4* (upregulated) (So and Croft, 2007; Pedersen and Lauritsen, 2009; Sakaguchi et al., 2013), as well as *S100a8* and *S100a9* (downregulated) (Lin et al., 2015) (Figure 3B).

We were not able to annotate T_H_17 cells because the transcript counts for T_H_17 gene markers, like *Rorc* (RORγt) or *Il17a* (IL-17A), did not meet the inclusion threshold. However, we found *Il21* (IL-21), one of the major cytokines secreted by T_H_17 cells, to be enriched in subcluster 2, alluding to the location where T_H_17 cells could be found (Supplementary Table). With this in mind, we looked at the DEG’s in normoxia and CH between subcluster 6 and subcluster 2 (Supplementary Material). However, other than enrichment in the GO term positive regulation of T-cell activation, none of the other pathways suggest changes in genes related to the T_H_17 cell function.

### Chronic hypoxia affects Treg and T_H_17 cell populations in the spleen

**FIGURE 4:**
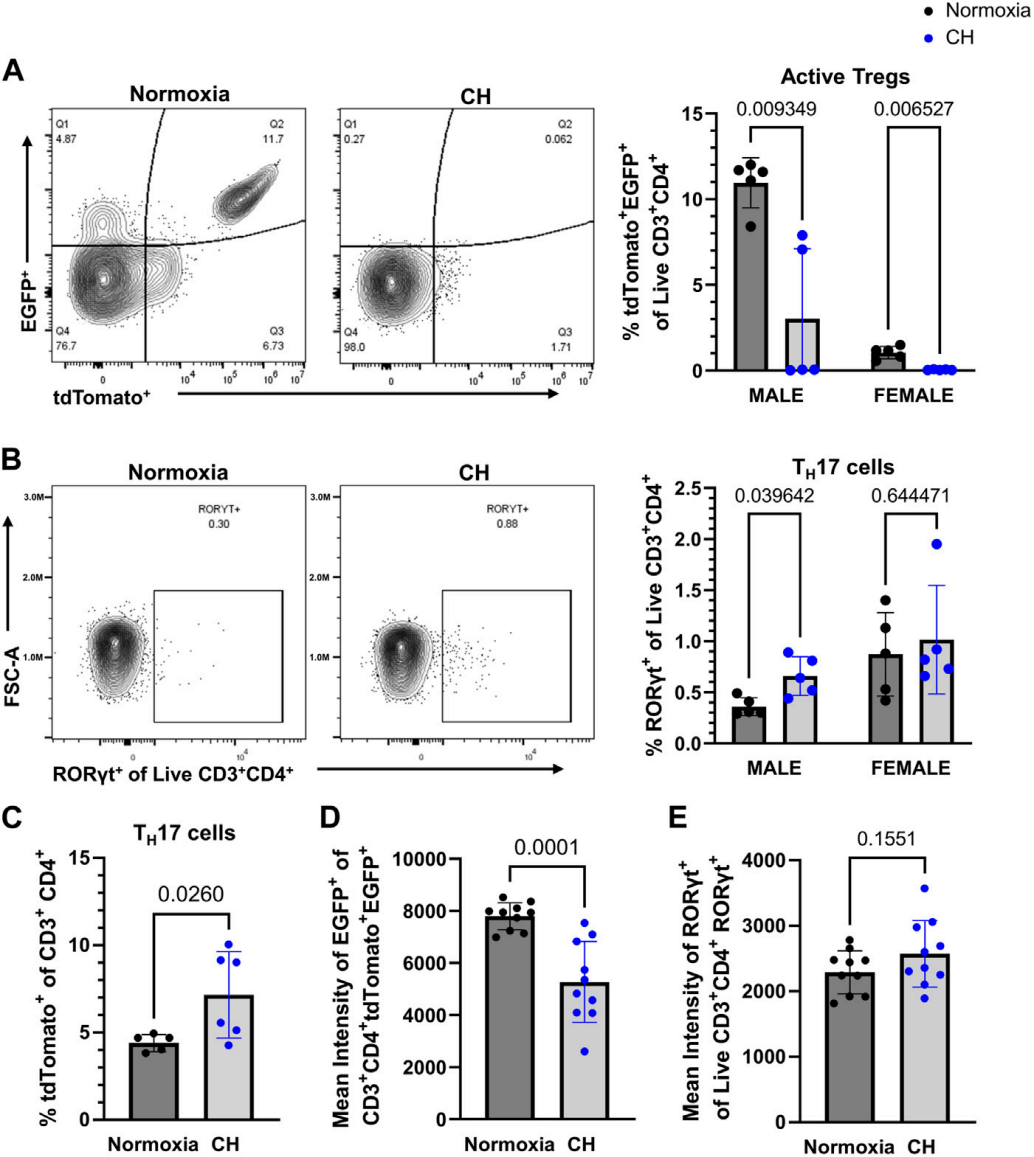
CH alters Tregs and T_H_17 cell proportions in the spleen. Foxp3^tm9(EGFP/cre/ERT2)Ayr^/J x Ai14-tdTomato or Il17a^cre^/J x Ai14-tdTomato male (*n* = 10) and female (*n* = 10) mice were exposed to normoxia (black) or 5-day CH (blue). Data show the following: **(A)** representative flow data and quantification of % active Tregs (tdTomato^+^EGFP^+^ of live CD3^+^CD4^+^ splenocytes), **(B)** representative flow data and quantification of % T_H_17 cells (RORγt^+^ of live CD3^+^CD4^+^ splenocytes), **(C)** % T_H_17 cells (tdTomato^+^ of CD3^+^ CD4^+^ splenocytes) from Il17a^cre^/J x Ai14-tdTomato mice (normoxia *n* = 5, 5-day CH *n* = 6), **(D)** combined data on the male and female mean intensity of EGFP, and **(E)** RORγt to assess FoxP3 and RORγt expression in Tregs and T_H_17 cells, respectively. Values are mean ± SD analyzed using multiple unpaired t-tests and Holm–Šidák correction.

Our group previously investigated the balance between Tregs and T_H_17 cells in CH, finding increased T_H_17 cells and no change of Tregs in the lungs of mice exposed to CH (Maston et al., 2017). To determine if CH affects Treg and T_H_17 cell populations in the periphery (outside of the lungs), flow cytometry was performed on splenocytes from Foxp3^tm9(EGFP/cre/ERT2)Ayr^/J x Ai14-tdTomato male and female mice exposed to normoxia or 5-day CH. Active Tregs, marked by tdTomato^+^EGFP^+^, were significantly decreased, following CH (Figure 4A). T_H_17 cells, marked by RORγt^+^, were significantly increased in males, following CH, although it was not significantly changed in females (Figure 4B). To further establish if T_H_17 cells increased, following CH exposure, we used the Il17a^cre^/J x Ai14-tdTomato mouse model (Hirota et al., 2011), which allows for fate mapping of IL-17A expression during T helper cell activation. We found increased CD3^+^ CD4^+^ tdTomato^+^ cells (IL-17 lineage), following CH exposure, in both males and females (Figure 4C).

We assessed the effect of CH on active FoxP3 expression by measuring the EGFP intensity in Tregs and found that both male and female mice had decreased expression levels of EGFP in Tregs, following CH exposure (Figure 4D). On the contrary, RORγt protein levels were not significantly different in T_H_17 cells, following CH exposure (Figure 4E).

### Tregs display markers of a less suppressive phenotype following CH

**FIGURE 5:**
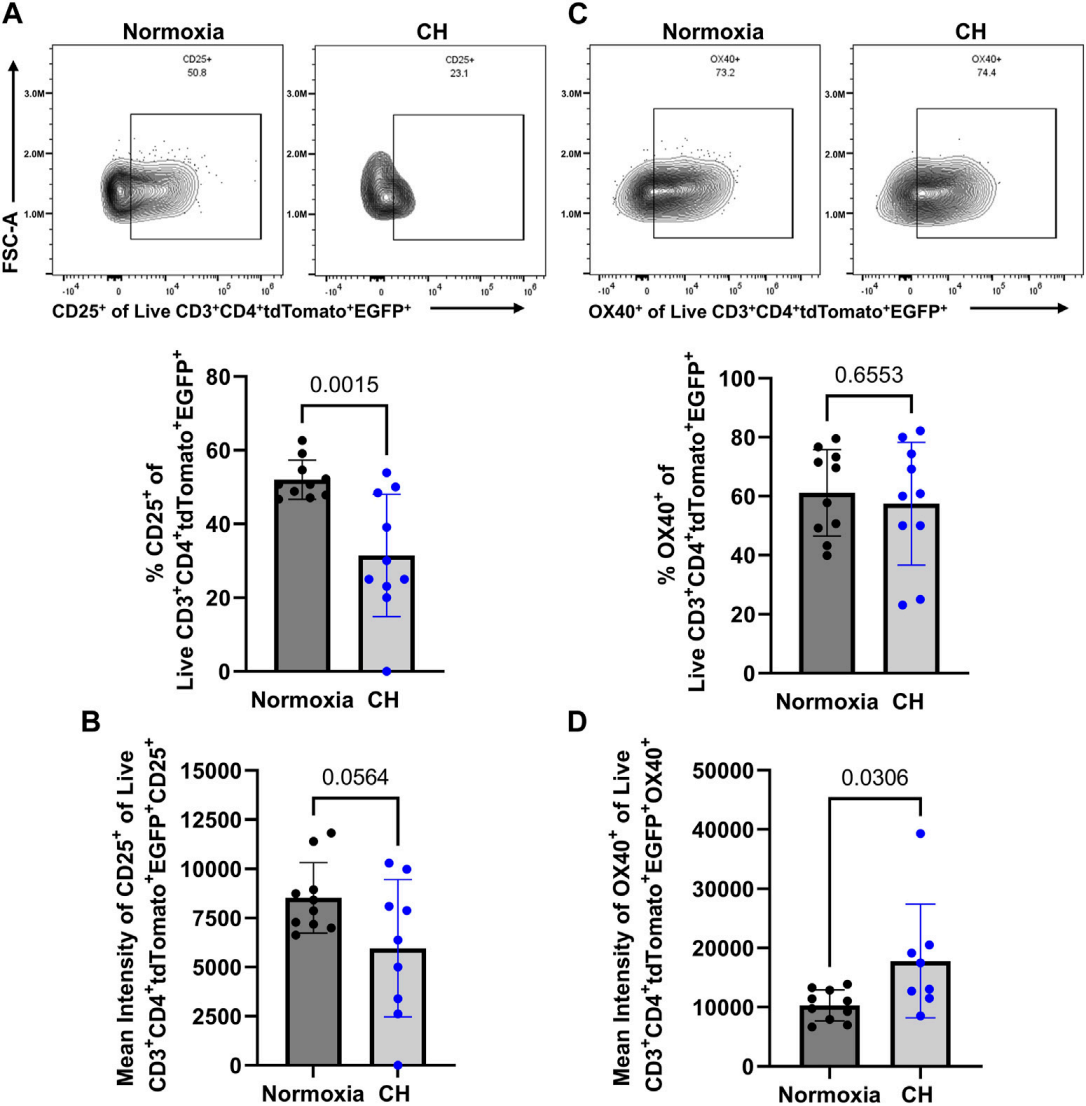
CH alters proteins associated with a less Treg suppressive capacity. Foxp3^tm9(EGFP/cre/ERT2)Ayr^/J x Ai14-tdTomato male (*n* = 10) and female (*n* = 10) mice exposed to normoxia (black) or 5 days of CH (blue). Data show the following: **(A)** representative flow data and quantification of % CD25^+^ CD3^+^ CD4^+^ tdTomato^+^ EGFP^+^ splenocytes, **(B)** mean intensity of CD25, **(C)** representative flow data and quantification of % OX40^+^ CD3^+^ CD4^+^ tdTomato^+^ EGFP^+^ splenocytes, and **(D)** mean intensity of OX40. Data from male and female mice were combined. Values are mean ± SD analyzed using multiple unpaired t-tests and Holm–Šidák correction.

After demonstrating a reduction in Tregs in the spleen and the amount of the EGFP (subrogate for FoxP3), following CH exposure, we aimed to determine whether Tregs show changes in suppressive markers at the protein level, following CH. Confirming the scRNA-seq data (Figure 3B), we found a decrease in the number of Tregs expressing CD25, following CH, and Tregs that expressed CD25 had a decreasing trend (*p* = 0.0564) in the amount of CD25 (Figures 5A, B). On the other hand, we found no effect of CH on the number of Tregs expressing OX40 but observed a significant increase in OX40 levels, following CH (Figures 5C,D).

### RORγt-expressing exTregs appear following CH

**FIGURE 6:**
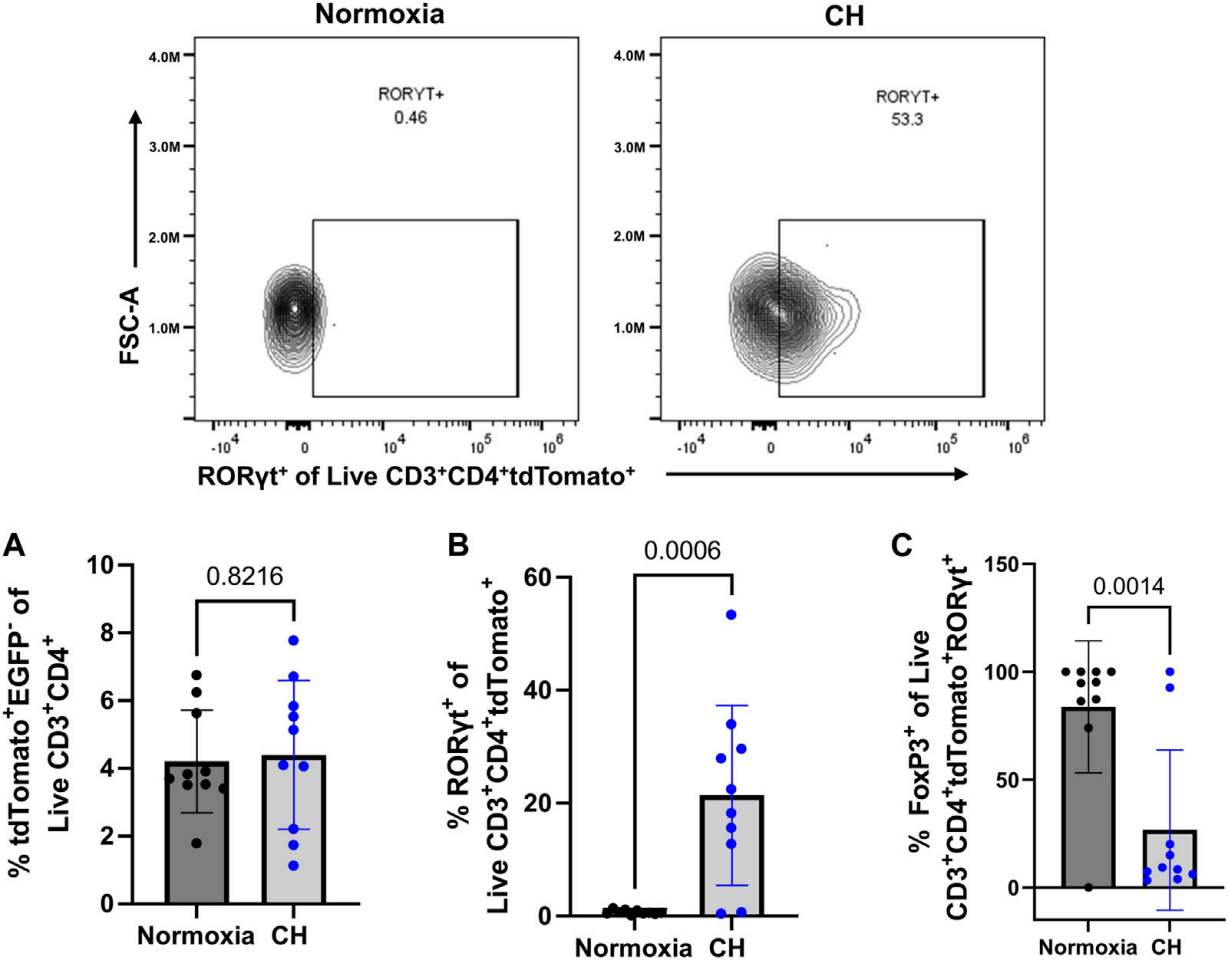
exTreg-T_H_17 cells appear following CH. Foxp3^tm9(EGFP/cre/ERT2)Ayr^/J x Ai14-tdTomato male (*n* = 10) and female (*n* = 10) mice exposed to normoxia (black) or 5-days CH (blue). Data show the following: **(A)** % of exTregs, **(B)** representative flow data and quantification of % exTreg-T_H_17 cells, and **(C)** % exTreg-T_H_17 cells that are FoxP3^+^. Data from male and female mice were combined. Values are mean ± SD analyzed using an unpaired *t*-test.

After discovering that Tregs displayed markers of a less suppressive phenotype following CH and increased T_H_17 cell numbers following CH, we aimed to determine if cells that were once Tregs, deemed exTregs, converted into exTreg-T_H_17 cells. To determine if CH caused an increase in the proportion of exTregs, the total number of tdTomato^+^EGFP^−^ within the live CD3^+^ CD4^+^ gate was determined. We found no significant change in the number of exTregs between normoxia and CH-exposed mice (Figure 6A). To determine if exTreg-T_H_17 cells exist under normal conditions, and if CH causes the differentiation of exTregs into cells expressing T_H_17 cell markers, we determined the proportion of RORγt^+^ exTregs. ExTreg-T_H_17 cells were minimal to non-existent under normal conditions (Figure 6B). We discovered that CH caused exTregs to express RORγt, the master transcription factor present in T_H_17 cells, suggesting that CH causes transcriptional reprogramming of exTregs into exTreg-T_H_17 cells (Figure 6B). In addition, we found that the few exTreg-T_H_17 cells present under normoxic conditions maintained FoxP3 expression, while a significant majority of exTreg-T_H_17 cells from CH lost their FoxP3 expression (Figure 6C).

### GO terms from the Treg subcluster highlight possible mechanisms for exTreg-T_H_17 cell conversion

**FIGURE 7:**
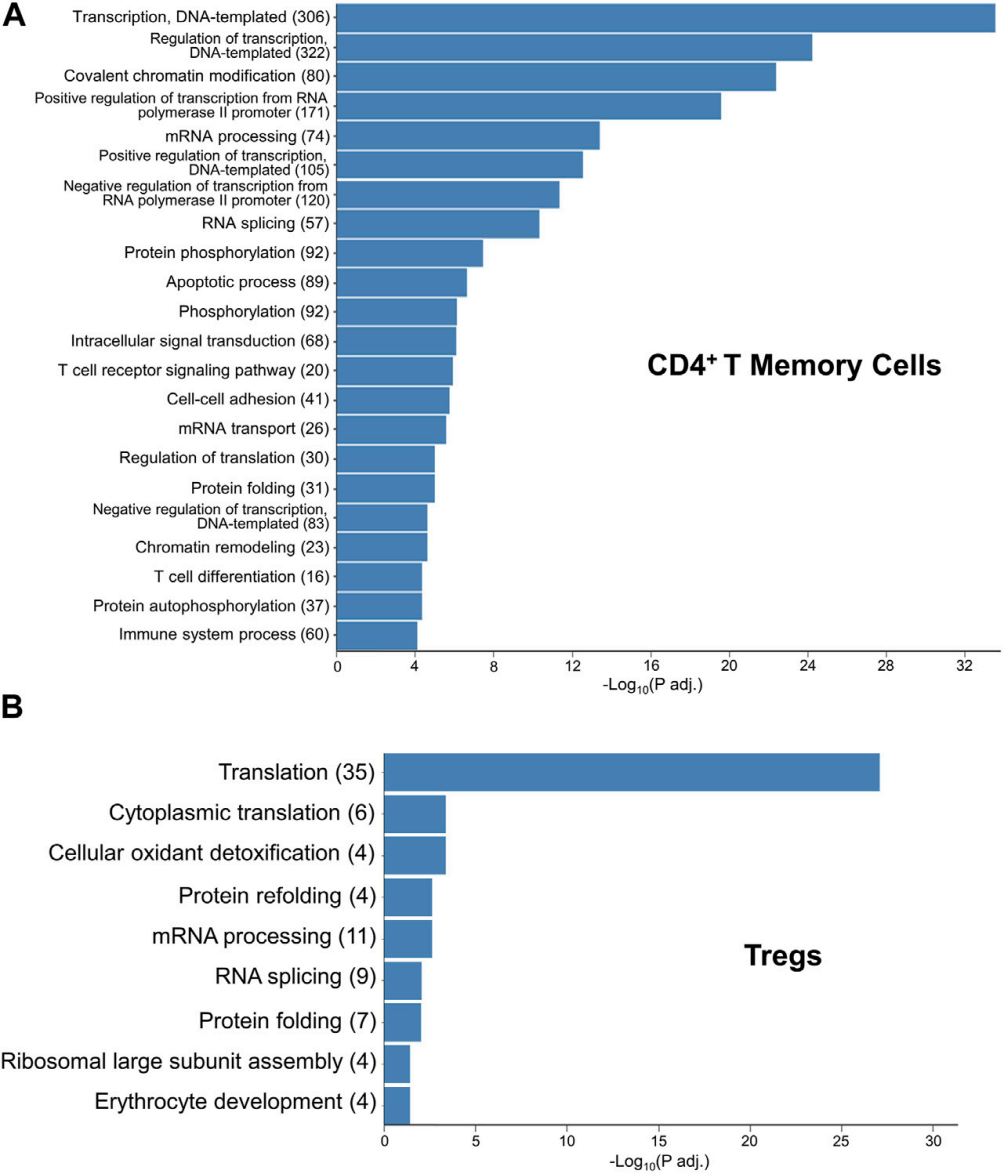
GO terms elucidating the pathways that likely contain the mechanism for hypoxia-induced exTreg-T_H_17 conversion. **(A)** Gene ontology enrichment in the CD4^+^ T memory cell cluster when comparing 5-day CH to normoxia using adj. *p*-value<0.05. **(B)** Gene ontology enrichment in the Treg cluster when comparing 5-day CH to normoxia using the adj. *p*-value ≤0.05.

To gain insights into how exTregs may convert into T_H_17 cells, following CH, we first explored the Gene Ontology (GO) pathways that were enriched within the CD4^+^ memory T-cell cluster when comparing CH to normoxia, finding the most abundantly enriched GO terms to be associated with DNA transcription, translation, and regulation; chromatin and protein modification; and apoptosis (Figure 7A). These pathways highlight large-scale changes occurring within the CD4^+^ memory T-cell cluster. We then explored the GO pathways enriched within the Treg subcluster when comparing CH to normoxia, once again finding pathways involved with DNA transcription and translation, as well as protein modifications, to be the most abundantly enriched (Figure 7B). Reactome data showed results similar to the GO pathways and other critical pathways, including regulation of apoptosis and cellular response to stress and starvation (Supplementary Table).

## Discussion

Pulmonary hypertension (PH) is defined as a mean pulmonary arterial pressure >20 mmHg at rest. PH due to chronic hypoxia (CH) is a multifaceted disease commonly caused by prolonged high-altitude exposure, sleep apnea, or chronic obstructive pulmonary disease (COPD) (Simon, 2010; Bauer et al., 2011; Negi et al., 2014; Kholdani et al., 2015; Bunel et al., 2019). Treatment options for CH-induced PH are limited to a handful of supportive therapies that do not prevent disease progression, the results of which lead to right heart failure and death (Hoeper et al., 2016; Hoeper et al., 2017; Tello et al., 2021). As previously stated, inflammation is a major contributor to elevated pulmonary pressure in CH-induced PH. Pulmonary inflammation observed in CH-induced PH occurs quickly, following CH exposure, and is present in the beginning stages of PH development (Maston et al., 2017). Our laboratory demonstrated the role of T_H_17 cells as the major pro-inflammatory driving force behind this inflammation (Maston et al., 2017). However, the role of Tregs and how the balance between Treg and T_H_17 cells outside of the lung is altered with CH exposure are not clear. This study demonstrates the novel finding that CH causes an imbalance between Tregs and Th17 cells by decreasing active Tregs and the appearance of exTreg-T_H_17 cells.

A decrease in the Treg suppressive ability has been closely tied to a loss of CD25 and a gain of OX40 expression (So and Croft, 2007; Pedersen and Lauritsen, 2009; Cabrera et al., 2012; Russell et al., 2012; Fu et al., 2020). When looking at current or active Tregs (EGFP^+^ tdTomato^+^), we found both the number of CD25^+^ Tregs and the expression level of CD25 within those cells to be decreased in CH, but only the expression level of OX40 in Tregs was increased by CH. Although we focused more heavily on *Il2ra* and *Tnfrsf4* and their corresponding proteins, CD25 and OX40, other DEGs also alluded to a reduction in the Treg suppressive ability. *S100a8* and *S100a9* were found to be downregulated in CH, both of which play an important role in Treg differentiation (Lin et al., 2015). The combination of altered Treg-suppressive genes and subsequent proteins, and decreased Foxp3 (EGFP reporter) expression along with the differentiation of exTregs to T_H_17 cells, following CH exposure, are most likely the causes for the diminished Treg cell count and increased T_H_17 cell count observed in CH.

Foxp3^tm9(EGFP/cre/ERT2)Ayr^/J x Ai14-tdTomato mice have lower populations of both EGFP^+^ tdTomato^+^ cells in female mice compared to male mice. This finding is consistent with reports demonstrating that female mammals produce fewer Tregs than males (Quintero et al., 2012; Nie et al., 2015). This finding may also explain the seemingly higher level of RORγt^+^ cells found in Figure 4B when comparing females to males.

We hypothesized increased exTreg numbers, following CH. Contrary to our hypothesis, we found no difference in the % of exTregs (tdTomato^+^EGFP^−^) between normoxia and CH. These data suggest a constant number of T cells that are exTregs. However, the conversion of exTregs into T_H_17 cells (exTreg-T_H_17 cells) was almost non-existent under normoxia, similar to the findings of other studies (Komatsu et al., 2014; Basdeo et al., 2015; Shevyrev et al., 2021); however, exTreg-T_H_17 cells were prevalent under hypoxia, demonstrating a hypoxia-dependent mechanism of phenotypic conversion or differentiation.

One limitation to this study is that the EGFP fluorescence intensity was lost with fixation. This meant we could not separate current or active Tregs from exTregs by using EGFP fluorescence when detecting intracellular markers, which require fixation and permeabilization. To circumvent this issue, we added a FoxP3 antibody to our panels, allowing us to track the current FoxP3 expression status.

The pathways found to be significantly altered using both GO and reactome give some insights into the possible mechanisms by which hypoxia causes exTreg to T_H_17 cell conversion, specifically those involved with protein translation, protein modifications, and cellular responses to stress. Furthermore, naïve CD4 T cells when cultured under T_H_17 skewing conditions in normoxia demonstrated accumulation of hypoxia-inducible factor 1α (HIF-1α), which was required for both RORγt and IL-17A upregulation, and hypoxia, which stabilizes and activates HIF-1α, further increased the proportion of T_H_17 cells under the same culture conditions. The authors further demonstrated that HIF-1α transactivates RORγt gene expression and targets Foxp3 for degradation simultaneously without affecting Foxp3 mRNA levels (Dang et al., 2011). However, this study did not explore whether these pathways control Treg and T_H_17 balance under *in vivo* hypoxia. We did not find HIF-1α or other HIF family members differentially expressed in Tregs or the CD4 T-cell memory cluster in scRNA-seq. However, it seems plausible that hypoxia-induced HIF-1α stabilization in exTregs could be the main driving force for exTreg-to-T_H_17 cell conversion through HIF-1α directly activating RORγt. This requires further investigation.

In conclusion, our study demonstrates that CH alters the balance between peripheral Tregs and T_H_17 cells by lowering the number of active Tregs while increasing T_H_17 cell numbers. Furthermore, our study establishes the emergence of exTreg-T_H_17 cells, following CH exposure. The contribution of these specific cells in the development of CH-induced PH remains to be elucidated, but they likely are part of a large component of the T_H_17 cell compartment. In addition, our study suggests therapies aimed to restore active Tregs and prevent the development of exTreg-to-T_H_17 cells show potential to treat CH-induced PH.

## Data Availability

The datasets presented in this study can be found in online repositories. The names of the repository/repositories and accession number(s) can be found in the article/Supplementary Material. This data can be found here: https://www.ncbi.nlm.nih.gov/geo/query/acc.cgi?acc=GSE219259.
