# Supplementary material for: Chronic hypoxia disrupts T regulatory cell phenotype contributing to the emergence of exTreg-TH17 cells
Source: Front Physiol. 2024 Jan 29;14:1304732. doi: 10.3389/fphys.2023.1304732 (PMC10859758; doi:10.3389/fphys.2023.1304732)
Supplement: Supplementary file 3 [file Table1.docx]

**Supplemental Material**

**Chronic Hypoxia Disrupts T Regulatory Cell Phenotype Contributing to the Emergence of exTreg-T_H_17 Cells**

**Benjamin J Lantz^*^, Mika Moriwaki, Olufunmilola M Oyebamiji, Yan Guo, Laura Gonzalez Bosc^*^**

**Correspondence*:**
Benjamin J. Lantz
bjlantz@salud.unm.edu

Laura Gonzalez Bosc, Ph.D.
lgonzalezbosc@salud.unm.edu

**Supplemental Table 2.** DEG CD4 memory T cell subclusters

**Supplemental Table 3.** DEG Major cell clusters

**Supplemental Table 4.** GO CD4 memory T cell subclusters and subcluster 6 Reactome

**Supplemental Table 5.** GO Major cell clusters

**Supplemental Table 6.** Normoxia Cluster 6 vs Cluster 2

**Supplemental Table 7.** CH Cluster 6 vs Cluster 2

GEO Accession Number: GSE219259
